# Osteogenic Activity of Collagen Peptide via ERK/MAPK Pathway Mediated Boosting of Collagen Synthesis and Its Therapeutic Efficacy in Osteoporotic Bone by Back-Scattered Electron Imaging and Microarchitecture Analysis

**DOI:** 10.3390/molecules181215474

**Published:** 2013-12-12

**Authors:** Hye Kyung Kim, Myung-Gyou Kim, Kang-Hyun Leem

**Affiliations:** 1 Department of Food & Biotechnology, Hanseo University, Seosan, Chungnam 356-706, Korea; 2 College of Korean Medicine, Semyung University, Chungbuk 390-711, Korea

**Keywords:** collagen hydrolysates, COL1A1 gene, ERK/MAPK, ovariectomized rats

## Abstract

Collagen hydrolysate (CH) has been reported to exhibit a positive effect on bone. In the present study, the *in vitro* effects of CH (<3 kDa) were examined and the *in vivo* experiments confirmed the positive effects of CH in ovariectomized (OVX) rats. Bone mineral density (BMD) was examined by DXA analysis. Scanning electron microscopic analysis and quantitative 3D-color backscattered electrons imaging analysis were performed on the lumbar vertebrae. CH increased osteoblastic cell proliferation and alkaline phosphatase activity in a dose-dependent manner. Collagen synthesis and collagen, type1, alpha1 (*COL1A1*) gene expression were also increased by CH treatment. Furthermore, CH-induced *COL1A1* gene expression was completely abolished by extracellular signal-regulated kinase (ERK) inhibitor, suggesting the involvement of ERK/MAPK signaling for transcriptional effects on *COL1A1* expression. OVX rats supplemented with CH showed osteoprotective effects as the BMD levels were increased compared with control. Moreover, CH prevented the trabecular bone loss induced by OVX and improved the microarchitecture of lumbar vertebrae. CH administration dose-dependently reduced the serum procollagen type I N-terminal propeptide level, which was elevated by OVX. The present study suggests that CH isolated in this study is a promising alternative to current therapeutic agents for the management of osteoporosis.

## 1. Introduction

Postmenopausal osteoporosis is characterized by low bone density and deterioration of bone microarchitecture, which leads to increased bone fragility and greater susceptibility to fractures [1]. Current treatments for osteoporosis are dominated by drugs that inhibit bone resorption although they also suppress bone formation that may contribute to the pathogenesis of osteonecrosis [2]. To restore the extensive bone loss, there is a great need for anabolic treatments that induce osteoblasts to build new bone. Pre-osteoblastic cells produce proteins of the extra-cellular matrix, including type I collagen at first, and then to successively produce alkaline phosphatase (ALP) and osteocalcin during differentiation to osteoblasts [3]. 

Mitogen-activated protein kinases (MAPKs) play important roles in cellular response to growth factors, cytokines, or environmental stress [4,5]. Extracellular signal-regulated kinase (ERK) is involved in cell proliferation/transformation and survival, whereas c-Jun N-terminal kinase is involved in stress responses. p38 MAPKs are involved in many cellular processes, such as inflammatory responses, cell cycle control and apoptosis [5,6,7,8]. Lai *et al.* [7] and Ge *et al.* [8] have suggested ERK is essential for osteoblast growth and differentiation. 

Collagen is one of the major structural proteins distributed throughout the whole body. Medically, collagen hydrolysates (CH) have long been used in pharmaceuticals and foods in the USA and Europe. Several studies suggest that CH improve bone collagen metabolism, bone mineral density, and bone mass content in rats and mice fed a calcium- or protein-deficient diet [9,10]. Oral administration of CH was also demonstrated to reduce bone loss by increasing the quantity of type 1 collagen and proteoglycan in the bone matrix of ovariectomized (OVX) rats [11], and by increasing the bone strength [12] and diameter of the cortical areas of femurs in OVX mice [13]. However, these studies used commercial CH products, and the effective doses of CH needed to ameliorate the osteopenia caused by OVX were relatively high (10–25 g/kg) [12,13]. In the present study, we prepared CH which exhibited functional effects from 150 and 500 mg/kg concentration based on lumbar vertebrae and whole body bone mineral density, respectively. Furthermore, CH enhanced collagen, type1, alpha1 (*COL1A1*) gene expression and collagen synthesis resulting in increased osteoblasts differentiation via the ERK1/2 MAPK signal transduction pathway. 

## 2. Results and Discussion

### 2.1. Hydrolysis Condition for CH and Osteogenic Activities of CH in MG-63 Cells

**Figure 1:**
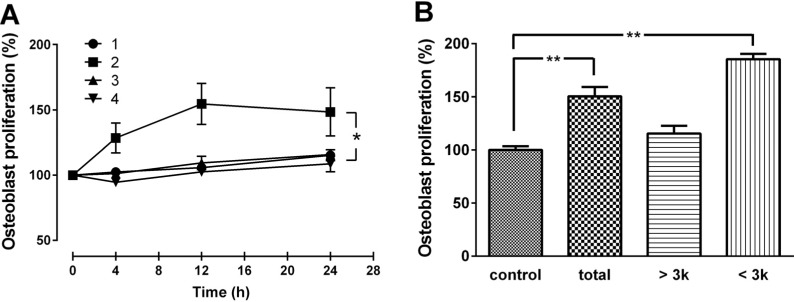
(**A**) Effect of CH produced by various enzyme combination and reaction time, and (**B**) various molecular size on MG63 cell proliferation. Cells were treated with various CH obtained by four different enzyme combinations [(1) alcalase + protamex; (2) protamex + flavourzyme; (3) flavourzyme + alcalase; (4) alcalase + protamex + flavourzyme] and three reaction times (4, 12, and 24 h) at 50 μg/mL concentration (**A**). The three different molecular sizes (total, >3 kDa, and <3 kDa) of CHs were tested for MG-63 cell proliferation (**B**). The data are expressed as a percentage of the control value, means ± SEM of the three cultures. * p < 0.05 *vs.* same time point. ** p < 0.01 *vs.* control.

Optimal hydrolysis conditions for the production of CH with the maximal osteoblast-like MG63 cell proliferation activity was examined by manipulating two reaction parameters; enzyme combination and reaction time. As shown in Figure 1A, among the four different combinations of enzymatic treatments, the combination of protamex+flavourzyme (PF) with 12 h reaction time provided the CH with the highest activity. Among the three fractions (total, >3 kDa, and <3 kDa), highest bone formation activity was observed with the small molecular weight (<3 kDa) CH (Figure 1B). Therefore, the enzyme combination of PF with a 12 h reaction time were chosen as the optimal conditions for digesting gelatin, and CH with molecular weight of <3 kDa was selected for further experiments. The average size of the CH was between 0.8 and 1.9 kDa, with a mean MW of 1.4 kDa, as determined by HPLC (data not shown).

**Figure 2:**
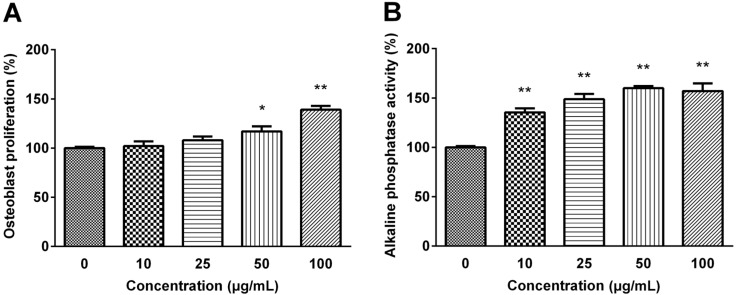
(**A**) Effect of selected CH on MG63 Osteoblastic cell proliferation (**B**) and MC3T3-E1 pre-osteoblastic cell alkaline phosphatase activity. The data are expressed as a percentage of the control value, means ± SEM of the three cultures. * p < 0.05 *vs.* control. ** p < 0.01 *vs.* control.

The osteogenic effects of selected CH were confirmed by osteoblast proliferation and differentiation. As shown in Figure 2, MG63 cell proliferation was dose-dependently increased exhibiting 1.2-fold of the basal value when cells were treated with 100 μg/mL CH (*p* < 0.01). 

The activity of ALP was examined to verify the effect of selected CH on the differentiation of pre-osteoblastic MC3T3-E1 cell. CH treatment significantly increased the ALP activity, one of the mature osteoblast phenotype markers, in a dose-dependent manner, and the ALP activity was increased to 160% of the control value with 50 μg/mL CH treatment (*p* < 0.01). 

### 2.2. Effects of CH on Collagen Synthesis and MAPK Signaling Pathway

**Figure 3:**
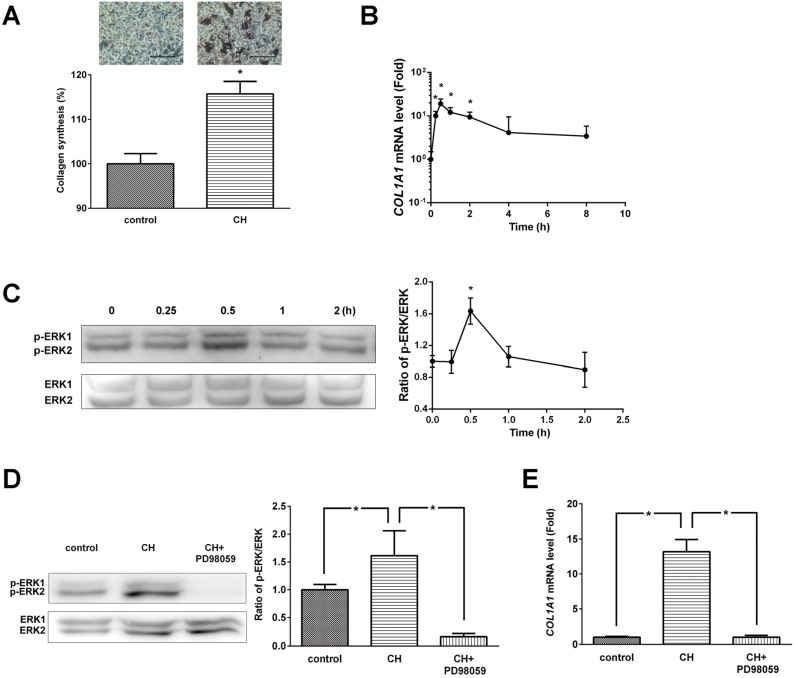
(**A**) Effects of CH on ERK/MAPK pathway mediated collagen synthesis and (**B**) *COL1A1* expression in MG63 cells. (**C**) Phosphorylation levels of ERK/MAPK and densitometric results of ERK phosphorylation. (**D**) ERK inhibitor (PD98059) abolished the CH-induced ERK phosphorylation. (**E**) ERK inhibitor (PD98059) inhibited the *COL1A1* gene expression. The data are expressed as a percentage (**A**), ratios of the control value (**D** and **E**), and ratios of the 0 h value (**B** and **C**). Data are expressed as means ± SEM of the three cultures. * p < 0.05 *vs.* control or 0 h.

Collagen content was determined by histochemical analysis using picro-sirius red staining (Figure 3A). CH (100 μg/mL) treatment significantly (*p* < 0.05) increased collagen synthesis. The exponential notation of *COL1A1* mRNA levels assessed by real-time PCR was significantly increased at 15 min, 30 min, 1 h, and 2 h (*p* < 0.05, Figure 3B). 

Several studies have indicated that ERK/MAPKs are essential in regulation of osteoblastic differentiation and maturation. Therefore, the activation statue of ERK signaling pathway was examined by western blot analysis. As shown in Figure 3C, CH treatment induced phosphorylation and activation of ERK1/2 within 0.5 h. The ratio of phosphorylated ERK/ERK protein expression at 30 min was significantly increased by CH treatment. 

We next examined the protein expression of CH-enhanced ERK activation in the presence of ERK inhibitor. As shown in Figure 3D, PD98059 completely abolished CH-induced phosphorylation of ERK1/2. CH treatment significantly increased the ratio of phosphorylated ERK/ERK protein expression (1.62 ± 0.44, *p* < 0.05) and the effect of CH was completely blocked by PD98059 treatment (0.16 ± 0.06, *p* < 0.05).

To further explore whether ERK signaling is necessary for CH-induced *COL1A1* gene expression, *COL1A1* mRNA levels were determined in the presence or absence of ERK inhibitor. CH treatment (100 μg/mL) dramatically increased *COL1A1* gene expression, and PD98059 completely blocked CH-induced *COL1A1* gene expression (Figure 3E).

### 2.3. Effects of CH in Osteoporotic Animal

**Figure 4:**
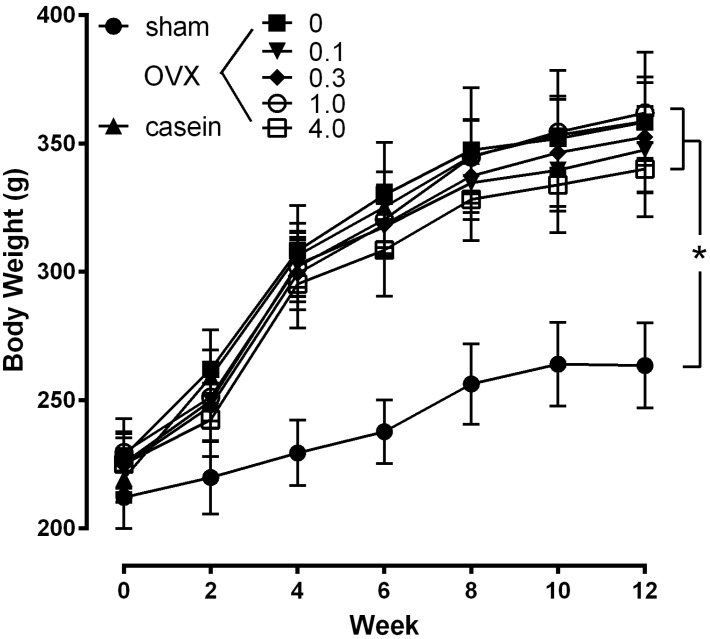
Effect of CH on body weight. The values represent the means ± SEM (*n* = 12 per group). sham: sham operated group; OVX: ovariectomized group administrated with 0%−4.0% CH; casein: OVX+casein (1%) group. * p < 0.05 *vs.* sham at the same time point.

Next, an *in vivo* experiment was carried out to verify the therapeutic efficacy of CH using a rat osteoporosis model. As shown in Figure 4, the body weight of the OVX and sham rats were not significantly different at the beginning of the study. Even though daily food consumption was not different in each group (data not shown), the body weight of the OVX rats were significantly increased after 2 wks post-operation compared with the sham group (*p* < 0.05). The body weight of the OVX group continued to be significantly higher than the sham group throughout the study, with no significant differences between OVX groups.

**Figure 5:**
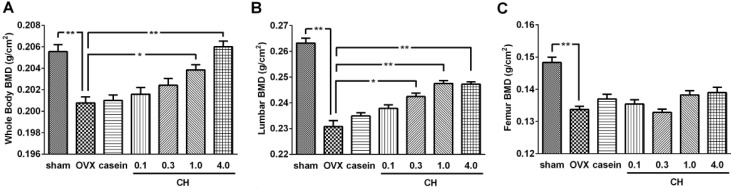
(**A**) Effects of CH on bone mineral density of whole body, (**B**) lumbar vertebrae (B; L1-L5), and (**C**) total femur. sham; sham operated group, OVX; ovariectomized control group. casein; OVX+casein (1%) group. 0.1, 0.3, 1.0, and 4.0; OVX rats administrated with 0.1%−4.0% CH. Data are expressed as means ± SEM.* p < 0.05 *vs.* OVX group. ** p < 0.01 *vs.* OVX.

Changes in BMD determined by DXA analysis were shown in Figure 5. The OVX significantly reduced the BMD of whole body (3.4%, Figure 5A), lumbar vertebrae (12.4%, Figure 5B), and femur (10.1%, Figure 5C) compared with the sham group (*p* < 0.01). Oral administration of CH dose-dependently improved whole body and lumbar vertebrae BMD while casein treatment failed to affect these BMD values. At the whole body level, CH exhibited significant effects from 1.0% concentration, whereas a significant effect from 0.3% was observed on the lumbar vertebrae (*p* < 0.05). The BMD of whole body reached a normal level with 4.0% CH treatment and lumbar vertebrae BMD was 94.0% of normal level with 1.0% CH treatment which it reached plateau. There were no significant effects on the femur BMD with CH or casein treatment.

**Figure 6:**
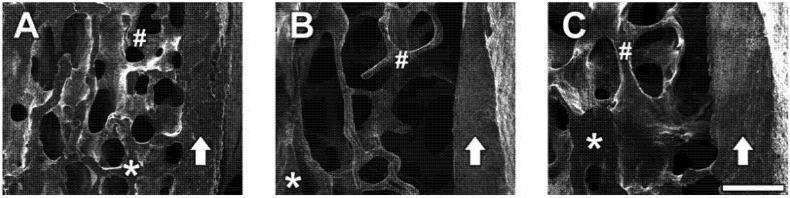
(**A**) Electron microscopic images of lumbar vertebrae in sham group, (**B**) OVX group, and (**C**) CH treated group. Secondary emission images of the lumbar vertebral body (centrum) which was cut sagittally on the half line. Arrow shows the cortical bone of the vertebral body. The asterisk area (*) is the cutting surface of cancellous bone. The trabecular bone areas (#) are placed on the left side of cortical bone (arrow). (**A**) Sham group showed densely packed space in the trabecular bone. (**B**) OVX group showed a thin trabecular bone (#) and the bone density was very low. (**C**) CH treated group showed a thicker trabecular bone compared with OVX and the bone density was higher than OVX. Scale bar = 200 μm.

Representative SE photomicrographs of each treatment group are shown in Figure 6. SE images revealed that OVX induced significant bone loss in trabecular bone areas (indicated as #) and reduced bone density (represented as dark grey color compared with the sham group) whereas the sham group showed densely packed spaces in trabecular bone. Administration of CH improved the micro-architecture of bones with thicker trabecular bone, increased bone density, and maintained the intactness and integrity of the trabecular bone, indicating its usefulness in the prevention of bone loss. However, cortical bone (indicated by an arrow) is not significantly affected by OVX (A; sham *vs.* B; OVX), while administration of 1.0% CH in OVX rats exhibited slightly thicker cortical bone (C), although this was not statistically significant. 

**Figure 7:**
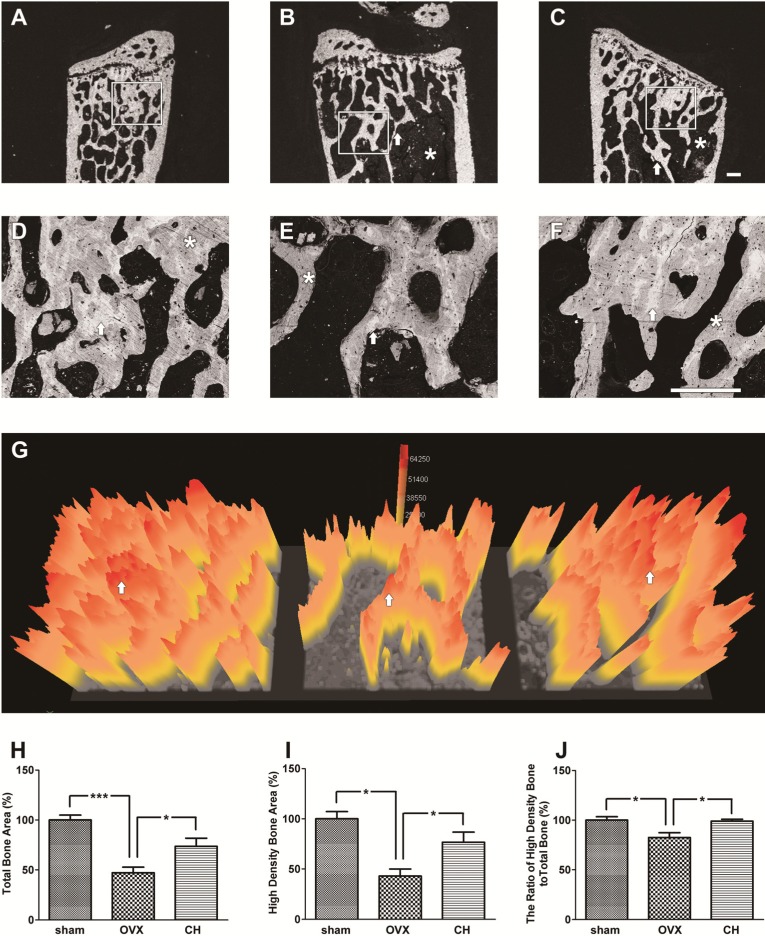
(**A**–**F**) Backscattered electron (BSE) microscopic images of lumbar vertebrae, and (**G**) 3D bone density map. (**A**, **D**) Sham group showed highly dense trabecular bone mass phenotype. (**B**, **E**) OVX group showed low trabecular bone mass phenotype. Arrow showed thinner trabecular bone and there was a massive empty space (*) in cancellous bone. (**C**, **F**) CH treated group showed a thicker trabecular bone compared with OVX and the empty space (*) was smaller than OVX. The **D**, **E**, and **F** were enlarged from the square areas of **A**, **B**, and **C**, respectively. Arrows and asterisks (*) in D, E, and F were the high density bone areas and the low density bone areas, respectively. Scale bar = 300 μm. (**G**) The image shows a bone density map. The high bone density area (white area pointed by arrows in **D**, **E**, and **F**) were represented as red color. Yellow color represented the total bone area. Arrows depicted in G were the same areas pointed by arrows in **D**, **E**, and **F**. (**H**) The ratio of total bone area in BSE images. (**I**) The ratio of high density bone area. (**J**) The ratio of high density bone area to total bone area. Data are expressed as means ± SEM. * p < 0.05 *vs.*OVX. *** p < 0.01 *vs.* OVX.

BSE microscopic images of the lumbar vertebrae bodies are illustrated in Figure 7. The D, E, and F were enlarged from the square areas of A, B, and C, respectively. As expected, there was a significant reduction of the trabecular bone mass in the OVX group (Figure 7B and E) compared with the sham group (Figure 7A and D) which showed highly dense trabecular bone mass. The arrows in Figure 7D and E show thinner trabecular bone and a massive empty space in the OVX group (Figure 7E) compared with the sham group (Figure 7D). CH treatment showed thicker trabecular bone (arrows in Figure 7F) and smaller empty space compared with the OVX group (Figure 7E). Contrast in a BSE image is dependent on the mineral density; high density areas are reflected as a bright grey color and low density areas are reflected as a dark grey color [14]. Sham group (Figure 7D) showed relatively bright grey color compared with the OVX group (Figure 7E), indicating loss of bone density by OVX. The CH treated group (Figure 7F) showed increased bright grey color compared with the OVX group (Figure 7E), indicating suppression of bone loss by CH treatment. 

The bone mineral density distribution was analyzed by quantitative BSE imaging (Figure 7D, E, and F) using a 3D color imaging technique [15,16]. OVX group (Figure 7G) showed reduced total bone area (indicated by yellow color) and high density bone area (indicated by red color) compared with the sham group, and CH treatment ameliorated these lumbar vertebrae bone structures. Calculated total bone area (Figure 7H) and high density bone area (Figure 7I) were decreased by 54.6% and 58.9%, respectively, by OVX compared with sham group. CH administration at 1.0% increased these parameters to 73.7% and 77.2% of control value, respectively. The ratio of high density bone to total bone (Figure 7J) was decreased by 17.6% by OVX and normalized by CH administration. 

Serum levels of bone turnover biomarkers including BSAP, PINP, OC, CTX, and CTX/TRACP 5b were measured as indicators of the protective effects of CH on osteoporosis in OVX rats (Table 1). As expected, OVX produced a significant increase in bone remodeling both in terms of resorption (CTX and CTX/TRACP index) and formation (BSAP, PINP, and OC). BSAP, OC, CTX, and CTX/TRACP levels were slightly increased by OVX, although there was no statistical significance, and CH administration dose-dependently decreased these parameters. Serum PINP concentrations were markedly increased in OVX rats and CH administration dose-dependently reduced the PINP level. There were no significant effects with casein treatment in all of the serum biomarkers. 

**Table 1:** Serum parameters of bone turnover. Values are means ± SEM (*n* = 12). * p < 0.05 compared with the OVX group. BSAP, bone-specific alkaline phosphatase; PINP, procollagen type I N-terminal propeptide; OC, osteocalcin; CTX, carboxyterminal telopeptide of collagen type 1; CTX/TRACP, ratio of CTX and tartrate-resistant acid phosphatase 5b concentration; sham, sham-operated group; OVX, ovariectomized group; CH50-CH2000, OVX groups treated with collagen hydrolysates (0.1%–0.4%, 50–2000 mg/kg); casein, OVX groups treated with casein (1%, 500 mg/kg).

Group	BSAP (U/L)	PINP (ng/mL)	OC (pg/mL)	CTX (ng/mL)	CTX/TRACP 5b index
Sham	9.96 ± 1.49	5.95 ± 0.93 *	2.76 ± 0.27	3.81 ± 0.79	0.49 ± 0.05
OVX	12.43 ± 1.22	17.28 ± 1.60	3.25 ± 0.34	4.07 ± 0.49	0.61 ± 0.08
CH50	16.34 ± 3.62	20.10 ± 2.76	2.80 ± 0.28	3.49 ± 0.84	0.44 ± 0.07
CH150	11.45 ± 1.68	14.32 ± 1.49	2.52 ± 0.37	3.17 ± 0.77	0.42 ± 0.05
CH500	10.25 ± 1.35	12.30 ± 1.09 *	2.42 ± 0.21	2.04 ± 0.35 *	0.25 ± 0.04 *
CH2000	10.27 ± 2.78	11.29 ± 1.35 *	2.02 ± 0.22 *	0.85 ± 0.18 *	0.09 ± 0.01 *
casein	12.53 ± 1.62	27.17 ± 2.15 *	2.94 ± 0.46	3.47 ± 0.62	0.40 ± 0.06

### 2.4. Discussion

This study demonstrated for the first time that CH (1.1~1.9 kDa) promoted osteoblast proliferation and differentiation via ERK/MAPK signaling pathway-mediated collagen synthesis. The bone-protective effects of CH with molecular weights <3 kDa isolated from porcine skin gelatin were investigated *in vitro* and *in vivo*. 

It was demonstrated that activation of ERK signaling pathways is a major promoter of osteoblast proliferation and differentiation [7,8]. In this study, CH stimulated the proliferation of the human osteoblastic cell line MG63 in a dose-dependent manner. The differentiation into osteoblasts represented by ALP activity was also dose-dependently promoted by CH treatment. Collagen content and the expression of *COL1A1* gene, involved in type 1 collagen synthesis, were also enhanced by CH treatment. Phosphorylation of ERK1/2 is increased by CH treatment. Furthermore, inhibition of ERK activation by PD98059 abolished the effects of CH on *COL1A1* gene expression suggesting ERK pathway involvement in osteogenic activity of CH.

We next investigated whether CH had a bone sparing/conserving effect under bone loss condition in a rat model of osteopenia. The bone growth in the experimental rats was evaluated based on BMD assessed by DXA, quantitative SEM, BSE and SE image analysis. One of the major determinants of bone strength is BMD, which predicts up to 60% of the variance of bone strength, and it best predicts the fracture risk in people without previous fractures [17]. In the current study, OVX significantly decreased the BMD of the whole body, lumbar vertebrae, and femur when compared with the sham group. Twelve weeks of CH administration reversed the loss of whole body and lumbar vertebrae BMD induced by OVX in a dose-dependent manner, whereas femur BMD was not affected by CH treatment. It is important to note that the lumbar vertebrae response with CH treatment was greater than that of the femur, suggesting its site preference of anabolic action. The bone loss is not uniform; cancellous bone is at a greater risk than cortical bone. Cancellous bone is the major component of vertebrae, and cortical bone is the chief component of the femur [18]. Therefore, the lumbar vertebrae play a critical role in predicting osteoporosis and monitoring the response to pharmacotherapy. It has also been reported that the density of femur is greater than lumbar vertebrae, and the volumetric density decrease with increasing age by 33% and 18% in lumbar vertebrae and femur, respectively [19]. These site differences of the lumbar vertebrae and femur may explain, at least in part, the greater restorative effects of CH on the lumbar vertebrae.

Deterioration of the trabecular architecture has been implicated in decreased bone strength and increased fracture incidence in humans. Thus, the assessment of trabecular architectural properties is necessary to evaluate the treatment impact on the quality of the lumbar vertebrae in addition to BMD. In this study, microarchitecture of the lumbar vertebrae assessed by SE, BSE, and 3D color imaging revealed that OVX dramatically reduced trabecular bone as well as total bone and high bone density area in trabecular bone. CH treatment counteracted the bone loss induced by OVX and preserved bone microarchitecture at 0.3%–1.0% dosage. These results indicate that CH contributes to the restoration of deteriorated trabecular architecture and lumbar morphology. 

It has been reported that because of estrogen deficiency, there was increase in body weight in OVX rats [20,21], and the increased body weight provides an additional stimulus for bone neoformation, serving as a partial protection against the osteopenia which occurs in long bones due to supporting the body weight [19]. In this study, none of the four dosage of CH (0.1%–4%) prevented the increase in body weight induced by OVX suggesting that CH at these dosages did not exert estrogen receptor agonist activity. Furthermore, the fact that CH administration did not influence the body weight of OVX rats indicates that bone density was not a result of a change in body mass.

The loss of bone mass and the deterioration of bone microarchitecture have been linked to an imbalance between bone formation and bone resorption. Biochemical markers of bone turnover have been widely used to measure the effects of various drugs on bone remodeling [22]. Serum BSAP, PINP, and OC levels are important biomarkers for assessment of bone formation rate, while TRACP 5b and CTX are major biomarkers for bone resorption rate [22]. However, it should be noted that OC is released into circulation both during bone formation and bone resorption. On the other hand, PINP is strictly released during the bone formation process, and is therefore more representative of bone formation than OC [22,23]. It has been reported that loss of bone mass induced by OVX may be accompanied by a significant increase in bone remodeling, as evidenced by enhanced levels of the bone turnover markers [24]. Post-menopausal osteoporosis and in OVX rats, the number of bone resorption sites, where elevated levels of the bone-resorption markers (e.g., CTX and TRACP 5b) can be detected, but also the number of bone-formation sites (e.g., PINP and OC) is increased [25]. A recent study demonstrated that secreted TRACP 5b is a reliable marker of osteoclast number and secreted CTX is a reliable marker of the resorbing activity of osteoclasts [26]. Osteoclast activity can be conveniently calculated by dividing the results obtained with a reliable marker of osteoclast activity (CTX) by the results obtained with a reliable marker of osteoclast number (TRACP 5b). Therefore, the ration of CTX/TRACP 5b appears to be an extremely useful parameter in the rat OVX model, where osteoclast activity is increased while osteoclast number is decreased [26]. Hence, CTX/TRACP 5b was used as an index of bone resorption. In our research, rats in the OVX group showed significantly reduced BMD of whole body and lumbar vertebrae (Figure 5) resulting from increased bone turnover, as indicated by higher levels of serum PINP and CTX/TRACP (Table 1) compared to the sham group. Administration of CH prevented the reduction in BMD, which was reflected by the reduction in serum PINP, OC, CTX, and CTX/TRACP indicating a reduction in bone turnover. The elevated bone turnover levels in OVX rats can mask additional effects of treatment with CH. Furthermore, measurements of serum markers are made at single time points and therefore subtle effects of treatments may not be reflected in levels of bone markers, despite the fact that changes are occurring in bone structure, as evidenced by BMD analysis. Therefore, the results from *in vitro* experiments exhibiting increased osteoblast proliferation, ALP activity, collagen synthesis, and *COL1A1* mRNA expression by CH treatment demonstrate more specific protective effect on bone.

Guillerminet *et al*. [12]. reported that *in vitro* osteogenic effect depends not only on collagen origin, but also on the molecular size of the CH. They suggested that collagen needs to be hydrolyzed to be able to interact with bone metabolism, and porcine origin commercial CH (2 kDa) was more effective than bovine or fish origin on osteoblast differentiation. They further demonstrated that 25 g/kg administration of CH (5 kDa) for 12 weeks significantly increased total body BMD and bone strength of OVX mice [12]. Same group also reported that ingestion of 25 g/kg of commercial porcine origin CH (average MW of 5 kDa, range 1–30 kDa ) for 1 month before OVX improved BMD of whole body [13]. Jackix *et al.* [11] suggested that 1.7 g/kg administration of commercial CH in the OVX rat protected the loss of vertebral mass and physical strength of the bone. The size and origin of the CH in their study were not elucidated. 

The monitoring of diet consumption in present study showed an intake of about 15.2 g/day. These amounts correspond to approximately 50 mg/kg BW of CH for 0.1% diet, 150 mg/kg for 0.3% diet, 500 mg/kg for 1% diet, and 2,000 mg/kg for 4% diet. In the present study, 0.3% and 1% treatment, which correspond to 150 mg/kg and 500 mg/kg, for 12 weeks showed significant effect on lumbar vertebrae and whole body BMD, respectively, in OVX rats. Lumbar vertebrae microarchitecture was also restored with 1% treatment, which corresponds to 500 mg/kg, indicating potently stronger effect than commercial CH used in other studies. It has been reported that various CHs differ with respect to their chemical composition of collagen fragments as well as by their pharmacological efficacy on human chondrocytes [27], and different CHs vary with respect to the width of MW distribution, average MW, and aggregation behavior [27]. Furthermore, porcine CH of 2 kDa was more effective than 5 kDa CH on *in vitro* bone formation/bone resorption activity [12]. Although we have not analyzed the amino acid composition of the CH, the potent effects of the CH used in the present study can be explained, at least in part, by the MW distribution (0.8~1.9 kDa) and average MW (1.4 kDa) difference. 

## 3. Experimental

### 3.1. Enzymatic Hydrolysis of Gelatin

Preliminary experiments were carried out to select two parameters; the optimal combination of proteases and enzyme reaction time for the production of the most effective CH on bone formation. Four combinations of enzymes (alcalase+protamex, protamex+flavourzyme, flavourzyme+alcalase, or alcalase+protamex+flavourzyme) and three reaction times (4, 12, and 24 h) were examined. Hydrated porcine skin gelatin (Geltec Co., Busan, South Korea) was hydrolyzed with various enzyme combinations (1%, w/w, Novozymes, Bagsvaerd, Denmark) at 50 °C for various times. The supernatants, after centrifugation at 3,000 *×g* for 15 min, were taken as the CH samples. The resultant 12 CHs were fractionated into 3 ranges of molecular weight (total, >3 kDa, and <3 kDa) using an Amicon ultra centrifugal filter (Centripep, Amicon, Beverly, MA, USA) and freeze-dried. 

### 3.2. HPLC

The range and the average of the molecular weight of the CH was determined by gel permeation chromatography-HPLC (Agilent 1200 series, Santa Clara, CA, USA). CH was dissolved in water at a concentration of 10 mg/mL and diluted with 30% acetonitrile containing 0.1% trifluoroacetic acid. The sample was centrifuged, filtered, and injected into a HPLC system with a Superdex Peptide column (30% CH_3_CN/0.1% trifluoroacetic acid, 0.5 mL/min). 

### 3.3. Osteoblastic Cell Proliferation and Alkaline Phosphatase Activity

The human osteoblast-like MG63 cells were obtained from the American Type Culture Collection (Rockville, MD, USA). The effects of various CH on proliferation of MG63 cell were determined by a colorimetric immunoassay kit (Roche Diagnostics, Mannheim, Germany) based on quantitating bromodeoxyuridine (BrdU) incorporation into the newly synthesized DNA. Alkaline phosphatase activity (ALP) was determined in pre-osteoblastic MC3T3-E1 cell using ALP B-test kit (Wako Chemical Co, Saitama, Japan). 

### 3.4. Collagen Content

Collagen synthesis was measured by the picro-sirius red method [28]. MG-63 cells were seeded in 12-well plates and allowed to adhere overnight. Osteogenic medium (DMEM with 10 mM β-glycerophosphate, 5 nM dexamethasone, and 50 μg/mL ascorbic acid) and CH (0 and 100 μg/mL) were added, and changed every 2–3 days. After 7 days, cells were fixed with Bouin’s fluid (8.3% formaldehyde and 4.8% acetic acid in saturated aqueous picric acid) for 1 h. Then the fixing solution was removed and the cells were stained with 0.1% sirius red (Direct Red 80, Sigma-Aldrich, St. Louis, MO, USA) in a saturated aqueous solution of picric acid for 30 min. The dye solution was removed and the cells were washed with 0.1 M HCl. Then, 0.5 M NaOH was added to dissolve the stained dye. The solutions were transferred to a 96-well plate and the absorbance was measured at 540 nm. 

### 3.5. Real-Time PCR

MG-63 cells were pretreated with or without an inhibitor of ERK (PD98059) and incubated with CH (100 μg/mL) for various time points (15 min–8 h). Total RNA was extracted using the RNeasy^®^ Protect Mini kit (Qiagen, Valencia, CA, USA), and cDNA was synthesized from mRNA using QuantiTect^®^ Reverse Transcription kit (Qiagen). Real-time PCR was performed using QuantiTectTM SYBR^®^ Green PCR kit (Qiagen) according to the manufacturer's protocol. The PCR primer sequences were as follows: collagen type 1 alpha 1 (*COL1A1*), forward 5'-GCG GCT CCC CAT TTT TAT ACC-3' and reverse 5'-GCT CTC CTC CCA TGT TAA ATA GCA-3'; and GAPDH, forward 5'-TCA TCA ATG GAA ATC CCA TCA CC-3' and reverse 5'-TGG ACT CCA CGA CGT ACT CAG C-3'. Analyses were performed using Rotor-Gene Q^®^ (Qiagen) and gene expression values were calculated based on the comparative ΔΔ CT method. 

### 3.6. Western Blot Analysis

Cells, pretreated with or without an inhibitor of ERK, were incubated with CH (100 μg/mL) for various times (15 min–2 h). Proteins were extracted with PRO-PREP Protein Extraction solution (Intron Biotechnology, Seongnam, Korea), aliquots of samples were subjected to 10% SDS-PAGE, and transferred onto a PVDF membrane. After blocking, the membrane was incubated with the primary antibody followed by incubation with secondary antibody conjugated with horseradish peroxidase. Immunoreactive bands were visualized using chemiluminescent imaging system (Fusion SL2, Vilber Lourmat, Marne-la-Vallée Cedex, France), and analyzed by the Bio1d software (Vilber Lourmat).

### 3.7. *In Vivo* Osteoporosis Model

All animals were treated according to the principles and procedures contained in the NIH Guide for Care and Use of Laboratory Animals with the approval of the Institutional Ethics Committee of Semyung University (smecae12-04-01). Female Sprague Dawley rats, 3-month old, were maintained at temperature 21–24 °C with a 12:12 h light-dark cycle. Bilateral ovariectomy (OVX) or sham operation was performed, and rats were divided into seven groups (*n* = 12 per group): sham-operated group (sham), OVX group (OVX), OVX-1% (w/w) casein group (casein), OVX-0.1% CH, OVX-0.3% CH, OVX-1% CH, and OVX-4% CH. Body weight (BW) was recorded once a week and food intake monitored daily. After 12 wks of feeding, bone mineral density (BMD) was measured and blood was collected by cardiac puncture. The lumbar vertebrae and femur were isolated and fixed in paraformaldehyde solution (Sigma-Aldrich Co.) for the quantitative scanning electron microscopy (SEM).

### 3.8. Assessment of BMD

Areal BMD was measured by dual energy X-ray absorptiometry (DXA, Discovery, Hologic Co., Bedford, MA, USA). The rats were anesthetized with Zoletil-Rompun mixture, and were placed in a horizontal position. From the scans, whole body, lumbar vertebrae (L1-L5) and total femur BMD was calculated using the bone mineral content of the measured area. 

### 3.9. SEM and Quantitative 3D BSE-SEM Imaging

Lumbar vertebrae (L4 or L5) were used for the secondary electron (SE) image and backscattered electrons (BSE) analysis (SEM scope, Quanta 400, FEI Co., Hillsboro, OR, USA). Contrast in a BSE image is dependent on the mineral density [14,29]. Therefore, BSE images were used for the quantitative 3D color imaging analysis for the measurement of mineral density distribution [17] using the Bio1d software (Vilber Lourmat, Marne-la-Vallée, France). Each image was classified into three levels: low, medium, and high density bone areas. The total bone areas were represented as yellow color and high bone density areas were represented as red color. The percentage of high density bone area was calculated by the portion of total bone area.

### 3.10. Biochemical Markers in Serum

Serum bone formation markers were assessed by the levels of bone-specific alkaline phosphatase (BSAP), procollagen type I N-terminal propeptide (PINP), and osteocalcin (OC) by a sandwich enzyme-linked immunosorbent assay (ELISA, Biomedical Technologies Inc., Stoughton, MA, USA). Bone resorption was evaluated by tartarate-resistant acid phosphatase 5b (TRACP 5b) concentrations using commercially available kits (Cusabio Co., Wuhan, China). β-isomer of serum C-telopeptide of type 1 collagen (CTX) was measured by an ELISA specific for rat CTX (RatLaps ELISA, IDS, Boldon, UK).

### 3.11. Statistical Analysis

Data were expressed as mean ± SEM. One-way ANOVA followed by Dunnett’s post-hoc test was used to determine statistical differences. *p* < 0.05 was considered to indicate statistical significance. 

## 4. Conclusions

In conclusion, a positive effect of CH, isolated in the present study, strongly support that CH is a promising alternative to current therapeutic agents for the management of bone formation and bone loss in postmenopausal osteoporosis.
